# Mesangial cells of lupus-prone mice are sensitive to chemokine production

**DOI:** 10.1186/ar2226

**Published:** 2007-07-07

**Authors:** Shuk-Man Ka, Chao-Wen Cheng, Hao-Ai Shui, Wen-Mein Wu, Deh-Ming Chang, Yu-Chu Lin, Ann Chen

**Affiliations:** 1 Department of Pathology, Tri-Service General Hospital, National Defense Medical Center, Cheng-Gung Road, Taipei 114, Taiwan, ROC; 2 Graduate Institute of Medical Sciences, National Defense Medical Center, Cheng-Gung Road, Taipei 114, Taiwan, ROC; 3 Department of Nutrition and Food Sciences, Fu-Jen Catholic University, Chung Cheng Road, Taipei County 242, Taiwan, ROC; 4 Division of Rheumatology/Immunology & Allergy, Department of Medicine, Tri-Service General Hospital, National Defense Medical Center, Cheng-Gung Road, Taipei 114, Taiwan, ROC

## Abstract

Infectious antigens may be triggers for the exacerbation of systemic lupus erythematosus. The underlying mechanism causing acceleration and exacerbation of lupus nephritis (LN) is largely unknown. Bacterial lipopolysaccharide (LPS) is capable of inducing an accelerated model of LN in NZB/W mice, featuring diffuse proliferation of glomerular resident cells. We hypothesized that mesangial cells (MCs) from LN subjects are more responsive to LPS than normal subjects. Cultured primary NZB/W and DBA/W (nonautoimmune disease-prone strain with MHC class II molecules identical to those of NZB/W) MCs were used. Monocyte chemoattractant protein-1 (MCP-1) and osteopontin (OPN) expressions either in the baseline (normal culture) condition or in the presence of LPS were evaluated by real-time PCR, ELISA, or western blot analysis. NF-κB was detected by ELISA, electrophoresis mobility-shift assay, and immunofluorescence. First, either in the baseline condition or in the presence of LPS, NZB/W MCs produced significantly higher levels of MCP-1 and OPN than the DBA/W MC controls. Second, NZB/W MCs expressed significantly higher levels of Toll-like receptor 4, myeloid differentiation factor 88, and NF-κB than the DBA/W MC controls, both receiving exactly the same LPS treatment. In conclusion, NZB/W MCs are significantly more sensitive than their normal control DBA/W MCs in producing both MCP-1 and OPN. With LPS treatment, the significantly elevated levels of both chemokines produced by NZB/W MCs are more likely due to a significantly greater activation of the Toll-like receptor 4-myeloid differentiation factor 88-associated NF-κB pathway. The observed abnormal molecular events provide an intrarenal pathogenic pathway involved in an accelerated type of LN, which is potentially infection triggered.

## Introduction

Lupus nephritis (LN) is a major complication of systemic lupus erythematosus and is associated with high rates of morbidity and mortality. Although clinical signs of renal involvement appear in only 50–80% of patients, the disease involves the kidney in almost all patients from whom sufficient tissue can be obtained for analysis [1]. Both renal and extrarenal events are involved in the pathogenesis of the disease. Bacterial and viral infections may serve as environmental triggers for the development or exacerbation of systemic lupus erythematosus in genetically predisposed individuals. Lupus patients are more prone to develop common (pneumonia, urinary tract infection, cellulitis, and sepsis), chronic (tuberculosis), and opportunistic infections, possibly because of genetic and immunologic defects [2]. Experimentally, when exposed to bacterial lipopolysaccharide (LPS), NZB/W mice promptly developed an accelerated diffuse proliferative nephritis [3,4], clinically and pathologically mimicking the transformation of renal lesion types from low grade to high grade in LN patients. Although increased immune complex deposition and proliferation of intrinsic cells in the affected glomeruli were observed in the NZB/W mice that received LPS administration [3], the actual mechanisms underlying the accelerated form of the LN model remain largely unclear. Recent studies suggest that mesangial cells (MCs) play a critical role in LN and regulate inflammatory responses inside the compromised glomeruli [5-9]. It is unknown whether MCs of the lupus-prone mice are more sensitive to an infectious agent than MCs of their normal control. This prompted us to evaluate chemokine production by NZB/W MCs both in the baseline (normal culture, 20% fetal bovine serum (FBS)) condition and when exposed to bacterial LPS to simulate (under 2% FBS), respectively, the normal physiological status and superimposed infection.

Recent studies have demonstrated that expression of LPS-induced monocyte chemoattractant protein-1 (MCP-1) [5] and of the osteopontin (OPN) [9] gene is NF-κB dependent. MCP-1, a CC chemokine, is mainly released by activated monocyte/macrophages, T cells, and natural killer cells, and attracts leukocytes and other mediators to sites of inflammation [6-8]. Cultured renal parenchymal cells, including MCs and renal tubular epithelial cells, produce MCP-1 in response to proinflammatory cytokines [10,11]. OPN is a chemotactic factor for monocytes and is an important mediator in glomerulonephritis [12-14]. OPN mRNA and protein are detected in cultured MCs subjected to a variety of stimuli [15,16]. Both of these chemokines play a crucial role in the pathogenesis of LN [17-19]. Toll-like receptor 4 (TLR-4) has been implicated in LPS signaling and is involved in the renal disease induced by expose to bacterial components [20-22]. TLR-4 mediates LPS signal transduction in collaboration with other molecules, such as CD14 and myeloid differentiation factor 88 (MyD88), resulting in rapid NF-κB activation [20,23].

In the present study, we demonstrated that NZB/W MCs (lupus-prone strain) produce significantly more chemokines than DBA/W MCs [24] (as the normal control; DBA/W mice are a nonautoimmune disease-prone strain with MHC class II molecules identical to those of NZB/W mice), both in the baseline (normal culture) condition and in response to LPS stimulation. This might explain how an infectious antigen triggers an accelerated and aggravated glomerular proliferative lesion in terms of intrinsic factors in the kidney.

## Materials and methods

### Primary culture of mesangial cells

Both female NZB/W and DBA/W F1 mice were obtained from the Animal Center of the College of Medicine of National Taiwan University and were maintained by the Animal Center of our National Defense Medical Center in a specific pathogen-free facility. All animal experiments were performed with the approval of the Institutional Animal Care and Use Committee of The National Defense Medical Center, Taiwan, and were consistent with the NIH Guide for the Care and Use of Laboratory Animals.

DBA/W F1 mice are a nonautoimmune disease-prone strain with MHC class II molecules identical to those of NZB/W mice. DBA/W mice do not spontaneously develop autoimmune disease; mice were monitored throughout their lifespan and show no proteinuria or detectable anti-double-stranded DNA or anti-DNA antibodies [24]. Aware that glomerulonephritis spontaneously develops in NZB/W mice beginning at 22–26 weeks of age, we chose to isolate glomeruli of female mice 7–8 weeks old for the following primary MC culture. Age-matched female DBA/W mice were used as the controls. The preparation of primary cultures of MCs was performed as described previously [25,26] with mild modification. Briefly, glomeruli were purified from minced renal cortex by serial sieving through meshes of different pore sizes, then the glomeruli suspension was digested for 20 minutes at 37°C with type IV collagenase, and the dissociated glomerular cells were cultured in RPMI 1640 medium containing 20% heat-inactivated endotoxin-free FBS, penicillin/streptomycin, and HEPES (10 mM) (GIBCO, Invitrogen, Carlsbad, CA, USA). The MCs show typical morphologic characteristics – positive for both α-smooth muscle actin and vimentin stain, but E-cadherin-negative as described previously [26]. The cultured MCs were used for experiments between passages 6 and 10.

### Experimental protocol

The cultured MCs were either plated in 20% FBS medium (baseline or normal culture condition) or arrested in 2% FBS medium for 2 hours, and were then incubated without or with 10 μg/ml LPS (*Salmonella minnesota *Re595; Sigma, St Louis, MO, USA) for mRNA analyses, protein analyses, and NF-κB activation assays at various time points. In the inhibition studies, cultured MCs were pretreated for 2 hours with a NF-κB inhibitor, *n*-tosyl-1-phenylalanine chloromethyl ketone or dexamethasone (both from Sigma), before addition of LPS to the cultures. The doses of pharmacological agents used were not cytotoxic for the cells as shown by the lactate dehydrogenase test.

### Proliferation assay

MCs were plated in 96-well plates. The cells were subsequently arrested in 2% FBS medium for 2 hours, and were then incubated without or with 10 μg/ml LPS for 6, 12, 24, or 48 hours. Methyl thiazoleterazolium (5 mg/ml; Sigma) was added (20 μl/well) and the mixture was incubated for 3 hours at 37°C. Dimethy-sulforide (Merck, Darmstadt, Germany) was then added (150 μl/well) for 15 minutes. The absorbance at 540 nm was determined using an ELISA plate reader (Bio-Tek, Burlington, VT, USA). The arithmetic mean optical density of six wells for each experimental point was used for cell proliferation levels.

### Real-time PCR

The total RNA was isolated from the MCs using TriZOL reagent according to the manufacturer's instructions (Invitrogen). For first-strand cDNA synthesis, 1.5 μg total RNA was used in a single-round reverse transcriptase reaction (total volume 25 μl), containing 0.9 μl Oligo(dT)_12–18 _primer, 1.0 mM dNTPs, 1 × first-strand buffer, 0.4 mM dithiothreitol, 80 U RNase out-recombinant ribonuclease inhibitor, and 300 U Superscript II RNase H (Invitrogen). Real-time PCR was subsequently performed in the ABI Prism 7700 Sequence Detection System (Perkin Elmer Applied Systems, Foster City, CA, USA) using the SYBR Green I PCR kit (Perkin Elmer Applied Systems). Each reaction contained 25 μl of 2 × SYBR green Master Mix, 300 nM primers, 5 μl of 1:10 dilution of the cDNA prepared above, and water to 50 μl. The reactions were then followed by 40 cycles of 30 seconds at 94°C, of 30 seconds at 60°C, and of 60 seconds at 72°C.

The primers used in this study were as follows: mouse β-actin, forward 5'-GACGGCCAGGTCACTAT-3' and reverse 5'-ACATCTGCTGGAAGGTGGAC-3'; mouse MCP-1, forward 5'-AGGTCCCTGTCATGCTTCTGG-3' and reverse 5'-ACAGTCCGAGTCACACTAGTTCA-3'; mouse OPN, forward 5'-CTCGTGCAGGAAGAACAGAAGC-3' and reverse 5'-GAGTCAAGTCAGCTGGATGAACC-3'; mouse TLR-4, forward 5'-CTCACAGATAGCCTGGCCAATC-3' and reverse 5'-CCATCTCACAAGGCATGTCCAG-3'; and mouse MyD88, forward 5'-ACTCCTTCATGTTCTCCATACC-3' and reverse 5'-ATCGAAAAGTTCCGGCGTTTGT-3'. The housekeeping gene β-actin was used as the internal standard.

### Immunocytochemistry and immunofluorescence

MCs were grown on glass slides and were fixed with 2% paraformaldehyde for 15 minutes. For immunocytochemistry, to study α-smooth muscle actin, vimentin, and E-cadherin, the sections were incubated overnight at 4°C with biotin-labeled mouse anti-α-smooth muscle actin (Neomarkers, Fremont, CA, USA), with goat antivimentin or anti-E-cadherin antibodies (Santa Cruz Inc., Santa Cruz, CA, USA), and then for 1 hour at room temperature with a streptavidin peroxidase system (DAKO, Carpinteria, CA, USA) or horseradish peroxidase-conjugated rabbit anti-goat antibody. Sections were counterstained with hematoxylin.

For immunofluorescence, to study NF-κB p65, the sections were incubated overnight at 4°C with rabbit anti-NF-κB p65 antibody (Abcam, Cambridge, MA, USA), and then for 2 hours at room temperature with fluorescein isothiocyanate-conjugated goat anti-rabbit IgG antibody (Cappel; Organon Teknika, Durham, NC, USA). The percentage of MCs showing nuclear NF-κB p65 was determined by counting at least 500 cells in each well under high power (×400) [13].

### Nuclear protein extraction

Nuclear extracts were prepared using a nuclear extract kit (Active Motif, Carlsbad, CA, USA) according to the manufacturer's instructions. The protein was measured using a Pierce BCA protein assay kit (Perbio Science, Etten-Leur, The Netherlands).

### Enzyme-linked immunosorbent assay

MCP-1 protein in culture supernatants was measured using commercial ELISA kits (Biosciences, Los Angeles, CA, USA) according to the manufacturer's instructions. The absorbance at 450 nm was determined using an ELISA plate reader (Bio-Tek). The MCP-1 protein expression levels at various time points were normalized to total protein as picograms per milligram.

Activation of the transcription factors, NF-κB p65 and activator protein-1, was measured in MC nuclear extracts using Trans-AM ELISA assay kits (Active Motif Europe, Rixensart, Belgium) according to the manufacturer's instructions. The absorbance was determined at 450 nm using an ELISA plate reader (Bio-Tek). Mouse recombinant NF-κB p65 (Active Motif Europe) was used as the standard to determine the concentration of NF-κB p65, and then the levels were normalized to nuclear protein as nanograms per milligram.

### Electrophoresis mobility-shift assay

For NF-κB activation, a nonradioactive electrophoresis mobility-shift assay kit was used according to the manufacturer's instructions (Panomics, Fremont, CA, USA). Six micrograms of nuclear protein were incubated for 30 minutes at room temperature with a biotinylated oligonucleotide containing the NF-κB-binding site, and then the samples were separated on a nondenaturing polyacrylamide gel (6%, with 2.5% glycerol) and blotted onto a Biodyne B (0.45 μm) positively charged nylon membrane (Pall Schweiz AG, Basel, Switzerland). The biotinylated nucleotides were detected using alkaline phosphatase-conjugated streptavidin and Chemiluminescent Reagent Plus (PerkinElmer Life Sciences, Boston, MA, USA) on film as described previously [27].

### Western blot analysis

For detection of cytoplasmic OPN, MCs were harvested and incubated for 20 minutes on ice in lysis buffer (20 mM Tris, pH 7.4, 137 mM NaCl, 10% glycerol, 1% Triton X-100, 2 mM ethylenediamine tetraacetic acid, and a protease inhibitor), and were then centrifuged at 14,000 rpm for 20 minutes. The protein concentration was determined with a BCA protein assay reagent (Perbio Science). The proteins were separated on a 10% SDS-PAGE gel and transferred to a polyvinylidene difluoride membrane (Millipore, Bedford, MA, USA), which was then incubated for 2 hours in 20 ml of 5% skim milk in Tris-buffered saline (0.05 M Tris-HCl, 0.9% NaCl, pH 7.4). The membrane was incubated overnight at 4°C with rabbit anti-OPN antibodies (Assay Designs, Ann Arbor, MI, USA) in Tris-buffered saline, and then, after three washes, for 1 hour at room temperature with horseradish peroxidase-conjugated goat anti-rabbit antibodies (Pierce, Rockford, IL, USA) in Tris-buffered saline. Bound antibody was detected using Chemiluminescent Reagent Plus (PerkinElmer Life Sciences) on film.

### Statistical analysis

All results are expressed as the mean ± standard error. Comparisons between two groups were made by an unpaired Student's *t *test. Differences among multiple groups were determined with one-way analysis of variance using Tukey's method for post-hoc analysis. *P *< 0.05 was considered statistically significant.

## Results

### mRNA expression of MCP-1 and osteopontin in the baseline (normal culture) condition

Under the baseline (normal culture, 20% FBS) condition, to determine whether the NZB/W MCs are 'hyperreactive' to chemokine production, the cells were plated in 20% FBS for 0–12 hours, and the levels of the chemokine transcripts were measured at the various time points. DBA/W mice are a nonautoimmune disease-prone strain with MHC class II molecules identical to those of NZB/W mice [24]. We used DBA/W MCs as the normal control throughout the experiment.

**Figure 1:**
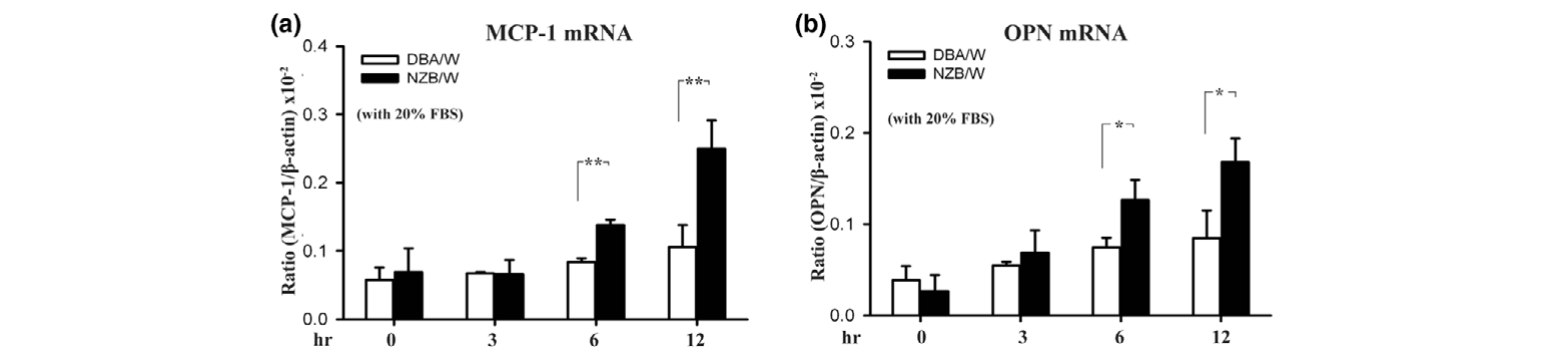
Monocyte chemoattractant protein-1 and osteopontin mRNA expression in NZB/W mesangial cells under the baseline condition. Mesangial cells were incubated in the baseline (normal culture, 20% fetal bovine serum (FBS)) condition for different periods of time, and then levels of **(a) **monocyte chemoattractant protein-1 (MCP-1) mRNA or **(b) **osteopontin (OPN) mRNA were examined by real-time PCR. The experiment was performed in triplicate, and results are expressed as the mean ± standard error, *n *= 6. *P < 0.05, **P < 0.01.

The levels of MCP-1 mRNA (*P *< 0.01) (Figure 1a) and of OPN mRNA (*P *< 0.05) (Figure 1b) at 6 and 12 hours were significantly higher in NZB/W MCs than in the DBA/W MC controls – although there was no significantly enhanced protein level of each of the chemokines of the NZB/W MCs, compared with the DBA/W MC controls. The latter effect could be due to the interference by serum (20% FBS) *per se *in the culture medium for protein extracted from the cultured cells or their supernatant.

The data suggest that NZB/W MCs are obviously hyperreactive in producing the two chemokines under the baseline (normal culture) condition.

### mRNA and protein expressions of MCP-1 and osteopontin under LPS stimulation

Cavallo and Granholm [3] demonstrated that the LPS-induced accelerated LN model in mice features significant proliferation of glomerular intrinsic cells, including MCs; however, the biological mode of action of the latter involved in the pathogenesis of the LPS-induced accelerated LN model remains largely unknown. To determine whether NZB/W MCs are capable of producing more MCP-1 and OPN than the DBA/W MC controls, when exposed to LPS (*S. minnesota*, the same source of LPS that was used to induce the accelerated LN model [3]) the cells were arrested in 2% FBS medium for 2 hours, and were then incubated with LPS (10 μg/ml) for 0–12 hours.

**Figure 2:**
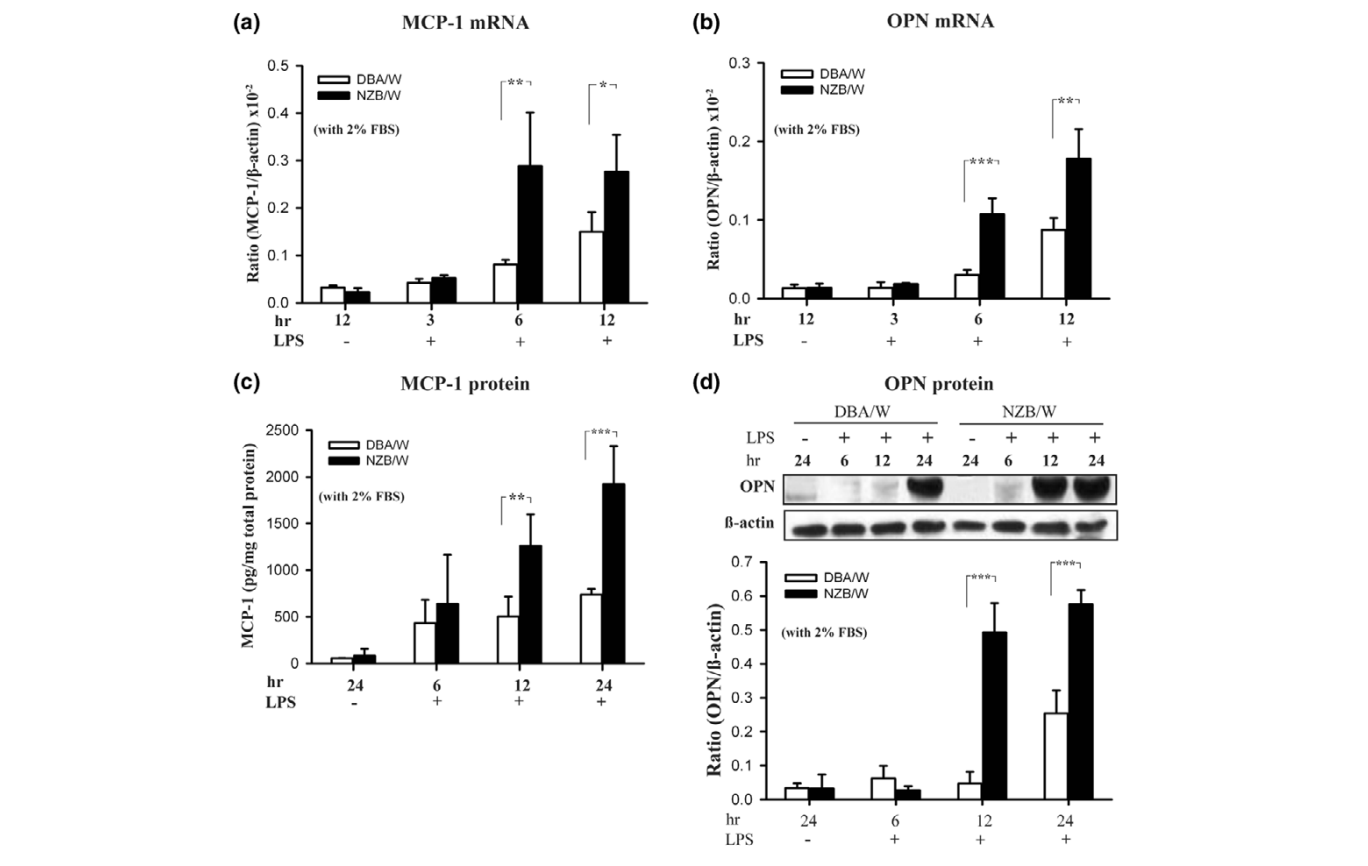
Monocyte chemoattractant protein-1 and osteopontin expression in NZB/W mesangial cells with lipopolysaccharide treatment. Growth-arrested (under 2% fetal bovine serum (FBS)) mesangial cells were incubated with 10 μg/ml lipopolysaccharide (LPS) for different periods of time, and then levels of **(a) **monocyte chemoattractant protein-1 (MCP-1) mRNA or **(b) **osteopontin (OPN) mRNA were examined by real-time PCR. **(c) **MCP-1 protein levels in the culture supernatant were measured by ELISA. **(d) **OPN protein levels in the cell lysate were measured by western blot analysis; semiquantitative data are shown. The MCP-1 protein expression levels at various time points were normalized to total protein (pg/mg). The experiment was performed in triplicate, and results are expressed as the mean ± standard error, *n *= 6. The last time point without LPS stimulation (unstimulated) was presented as the negative control. *P < 0.05, **P < 0.01, ***P < 0.005.

Our preliminary data showed that the concentration of LPS was not cytotoxic throughout the experiment. First, the real-time PCR showed an increase in MCP-1 mRNA levels after 6 or 12 hours (Figure 2a) and in OPN mRNA levels at 6 or 12 hours (Figure 2b) of LPS treatment in NZB/W MCs, and these increases were significantly higher than those in the identically treated DBA/W MC control (*P *< 0.05). Second, the ELISA showed significantly higher MCP-1 levels in the medium of NZB/W MC cultures compared with DBA/W MC cultures after 12 or 24 hours of incubation with LPS (*P *< 0.01) (Figure 2c). Western blot analysis also showed greatly enhanced levels of OPN in NZB/W MCs compared with the DBA/W MC controls at the same times (both, *P *< 0.005) (Figure 2d).

These data indicate that MCs from NZB/W mice (lupus-prone strain) are more sensitive than the DBA/W MC controls to LPS stimulation.

### Involvement of the TLR-4-MyD88 pathway in mesangial cells treated with LPS

**Figure 3:**
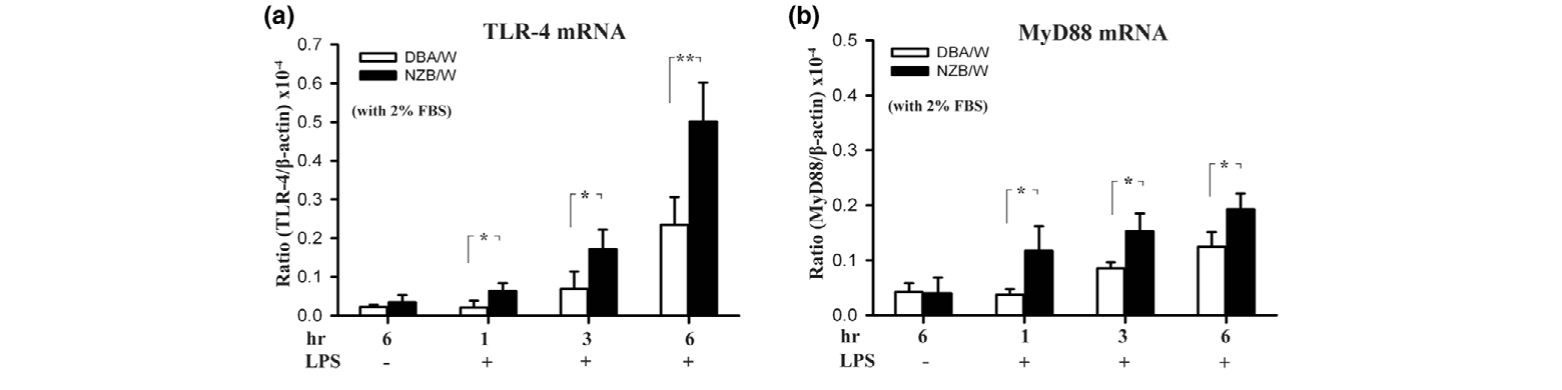
Toll-like receptor 4 and myeloid differentiation factor 88 mRNA in NZB/W mesangial cells (lipopolysaccharide treatment). Growth-arrested (under 2% fetal bovine serum (FBS)) mesangial cells were incubated with 10 μg/ml lipopolysaccharide (LPS) for different periods of time, and then Toll-like receptor 4 (TLR-4) and myeloid differentiation factor 88 (MyD88) mRNA levels were measured by real-time PCR analysis. The experiment was performed in triplicate, and results are expressed as the mean ± standard error, *n *= 6. The last time point without LPS stimulation (unstimulated) was presented as the negative control. *P < 0.05, **P < 0.01.

The TLR-4 pathway has recently been shown to act as a signal transducer for LPS in various tissues, resulting in cellular activation and the release of cytokines, chemokines, reactive oxygen species, and nitric oxide [20,28,29]. As shown in Figure 3, up-regulation of both the TLR-4 and MyD88 transcripts was observed in the NZB/W MCs after 1, 3, or 6 hours of LPS stimulation, compared with the DBA/W MC controls that were identically treated (each, *P *< 0.05).

### Involvement of NF-κB activation in mesangial cells treated with LPS

Phosphorylation of NF-κB p65 is required for optimal induction of the NF-κB target genes in response to a variety of proinflammatory stimuli, including MCP-1 [5] and OPN [9]. We evaluated whether the NF-κB p65 activation occurred in NZB/W MCs under LPS stimulation, and compared it with that in the DBA/W MC controls using immunofluorescence, the electrophoresis mobility-shift assay, and the ELISA.

**Figure 4:**
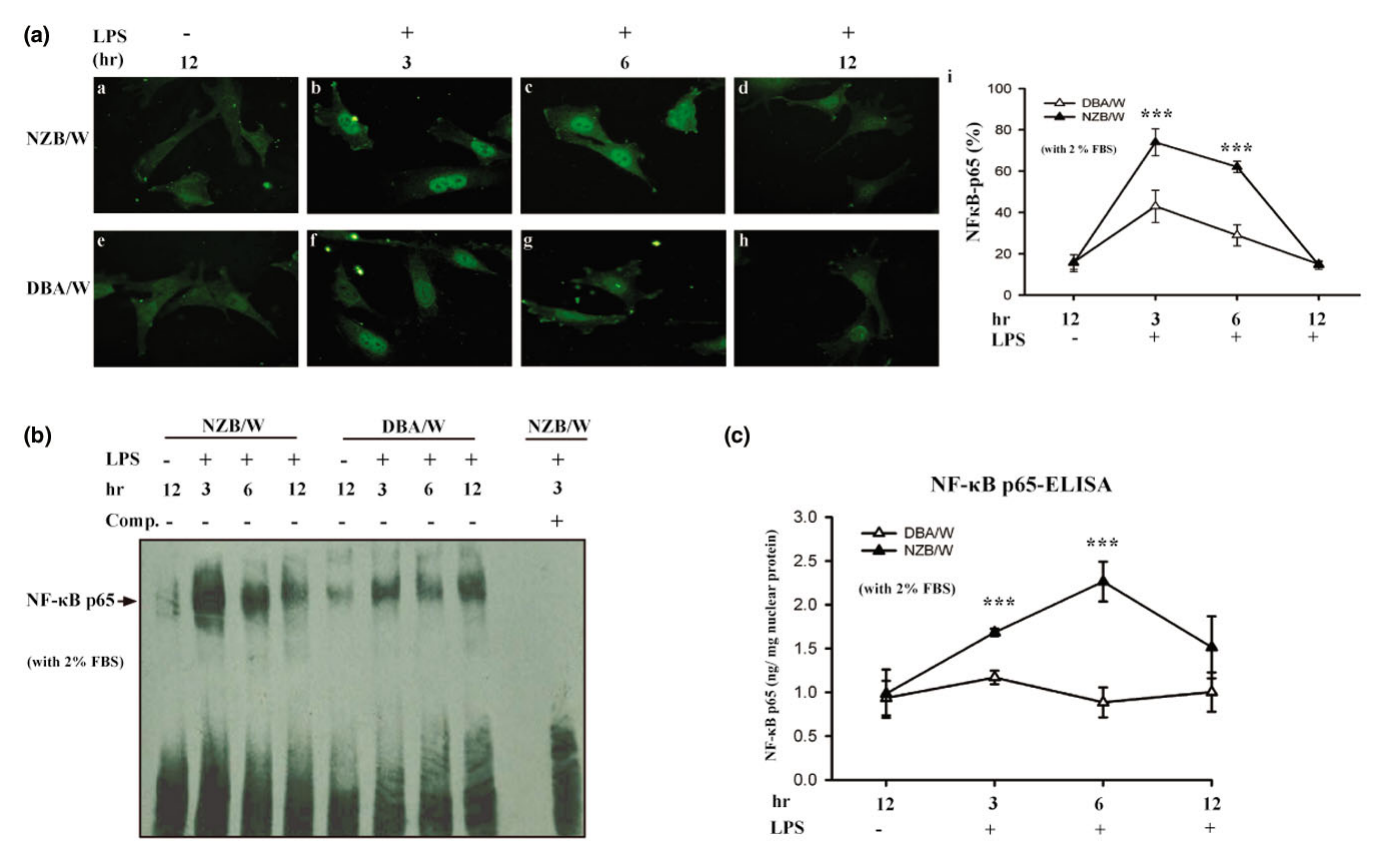
NF-κB p65 activation in NZB/W mesangial cells with lipopolysaccharide treatment. Growth-arrested (under 2% fetal bovine serum (FBS)) mesangial cells (MCs) were incubated with 10 μg/ml lipopolysaccharide (LPS) for different periods of time, and then the distribution of NF-κB p65 was examined. **(a) **Immunofluorescence: images a–d, NZB/W MCs; images e–h, DBA/W MCs (nonimmune strain, served as control) incubated for 0–12 hours with LPS; and image i, semiquantitative data. **(b) **Electrophoresis mobility-shift assay performed using a DIG-labeled synthetic oligonucleotide and nuclear extract from MCs. The competition assay used the same unlabeled oligonucleotide at a 10-fold higher concentration. Arrow, NF-κB p65 binding bands. Comp., the abbreviation of competition. **(c) **ELISA performed using the TransAM NF-κB p65 kit. The NF-κB p65 expression levels at various time points were normalized to nuclear protein (ng/mg). The experiment was performed in triplicate, and results are expressed as the mean ± standard error, *n *= 6. The last time point without LPS stimulation (unstimulated) was presented as the negative control. ***P < 0.005 versus DBA/W MC controls.

First, as shown in Figure 4a, the NF-κB p65 was markedly present in the nuclei of the NZB/W MCs after 3 and 6 hours (Figure 4, images a-c and i; *P *< 0.005), respectively, of LPS stimulation, and then fell to basal levels at 12 hours (Figure 4a, images d and i). In contrast, the DBA/W MC controls (Figure 4a, images f-h and i) showed only little NF-κB p65 in their nuclei after incubation with LPS.

Second, the electrophoresis mobility-shift assay further confirmed the activation of NF-κB p65 by showing significantly stronger NF-κB DNA binding in NZB/W MCs than in DBA/W MC controls, after 3 and 6 hours of LPS stimulation (Figure 4b). Using the ELISA, again NZB/W MCs showed significantly higher levels of nuclear NF-κB p65 than those of the DBA/W MC controls (1.68 **± **0.04 or 2.26 **± **0.22 ng/mg nuclear protein compared with 1.16 **± **0.07 or 0.88 **± **0.17 ng/mg nuclear protein after 3 or 6 hours, respectively; *P *< 0.005) under LPS treatment (Figure 4c).

Third, a transcription factor – activator protein-1, sometimes together with NF-κB – is involved in a variety of signaling pathways [9]. The ELISA confirmed that only low levels of activator protein-1, however, were detectable in both of the MCs, and there was no significant difference between them (data not shown).

**Figure 5:**
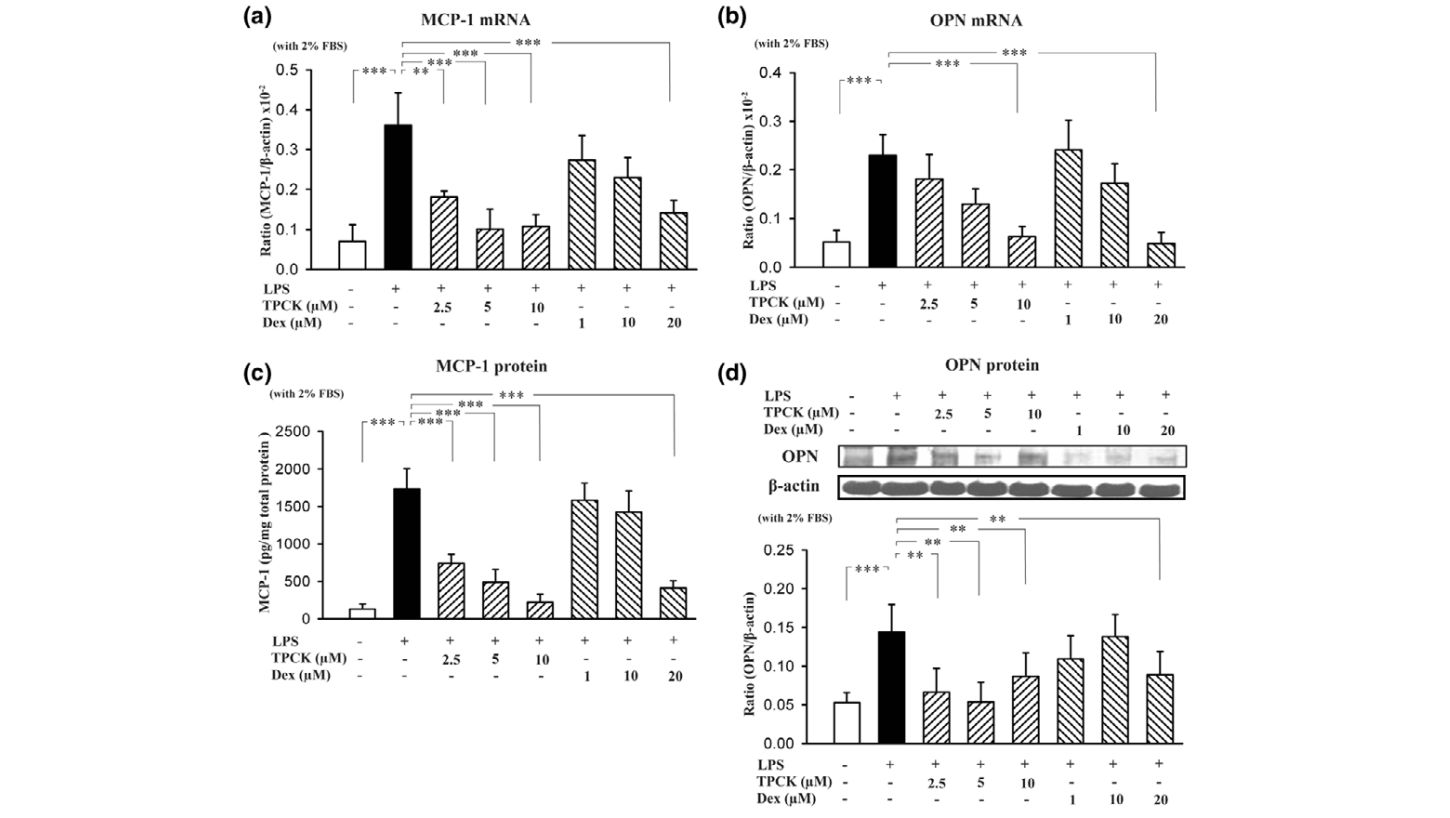
NF-κB inhibitor effect on lipopolysaccharide-induced monocyte chemoattractant protein-1 and osteopontin expression in NZB/W mesangial cells. Growth-arrested (under 2% fetal bovine serum (FBS)) mesangial cells were preincubated for 2 hours with *n*-tosyl-1-phenylalanine chloromethyl ketone (TPCK) or dexamethasone (Dex), and then 10 μg/ml lipopolysaccharide (LPS) was added for 12 hours: **(a) **monocyte chemoattractant protein-1 (MCP-1) mRNA levels and **(b) **osteopontin (OPN) mRNA levels were measured by real-time PCR analysis. Growth-arrested (under 2% FBS) mesangial cells were preincubated for 2 hours with TPCK or Dex, then 10 μg/ml LPS was added for 24 hours: **(c) **MCP-1 protein levels in the supernatant were measured by ELISA, and **(d) **OPN protein levels in the cell lysate were measured by western blot analysis; semiquantitative data are shown. The MCP-1 protein expression levels at various time points were normalized to total protein (pg/mg). The experiment was performed in triplicate, and results are expressed as the mean ± standard error. **P < 0.01, ***P < 0.005, *n *= 6. White bar, absence of LPS; black bar, LPS alone; hatched bar, TPCK and LPS or Dex and LPS.

Finally, NZB/W MCs were preincubated for 2 hours with *n*-tosyl-1-phenylalanine chloromethyl ketone (2.5–10 μM) or with dexamethasone (1–20 μM) (both as NF-κB inhibitors), and were then subjected to the same LPS treatment (12 hours for mRNA analysis and 24 hours for protein analysis, respectively) as that mentioned above. All concentrations of *n*-tosyl-1-phenylalanine chloromethyl ketone and the highest concentration of dexamethasone inhibited the LPS-induced increase in MCP-1 mRNA (Figure 5a) and in OPN mRNA (Figure 5b) levels in NZB/W MCs and the LPS-induced increase in protein levels of MCP-1 (ELISA; Figure 5c) and of OPN (western blot analysis; Figure 5d).

In summary, the data show that, under LPS stimulation, NZB/W MCs exhibited a significantly augmented activation of the TLR-4-MyD88-NF-κB signaling pathway, and its resultant significantly enhanced production of MCP-1 and of OPN, compared with the DBA/W MC controls.

## Discussion

Cavallo and Granholm [3,4] established a LPS-induced accelerated LN model in NZB/W mice. In the present study, we detected chemokine productions by NZB/W MCs both in the baseline (normal culture, under 20% FBS) condition and when exposed to bacterial LPS (under 2% FBS), respectively, and we performed mechanistic experiments to dissect the potential mechanisms responsible for the events.

Glomerular infiltration of monocytes/macrophages is frequently observed in a variety of human LN [30,31] and the chemokine most commonly involved in renal monocytes/macrophages recruitment is MCP-1 [19,32]. OPN is a chemoattractant and has also been shown to recruit monocytes into the interstitium of the kidney in LN [17,33]. Both the chemokines have been identified as highly expressed in MCs under disease conditions [10,34].

In the present study, we first demonstrated that NZB/W MCs are hyperreactive to generate MCP-1 and OPN in the baseline (normal culture, 20% FBS) condition. We also observed that NZB/W MCs expressed much higher levels of IL-6 and inducible nitric oxide synthase mRNAs than the DBA/W MC controls in the baseline (normal culture, 20% FBS) condition, although there was no significant difference in mRNA levels of IL-1β, IL-4, IL-12 or TNFα between NZB/W MCs and DBA/W MCs (unpublished data). We believe that NZB/W MCs of the lupus-prone mice are very likely to be 'hypersensitive' in their physiological growth status. The mechanisms responsible for the NZB/W MCs being obviously hyperreactive in producing the two chemokines (OPN and MCP-1) under the baseline condition need further investigation, although we thought that the complicated genetic defect of lupus-prone mice could be one of the potential mechanisms responsible for this particular property. On the other hand, it has been observed that MCP-1 mRNA levels are increased in LPS-induced renal tubular cell injury [5] and in urinary sediment of lupus patients [30], reflecting LN activity. OPN, which similarly acts both as a chemokine and as a cytokine, is detected in the glomerular crescents of severe forms of experimental glomerulonephritis [12,13] and in human LN [14]. Similarly, we found that NZB/W MCs are also sensitive to producing significantly greater levels of chemokines (MCP-1 and OPN) in response to LPS stimulation (under 2% FBS) than the DBA/W MC controls. These findings support the observations that an environmental infectious agent, LPS, may contribute to the exacerbation of LN [2,35].

We then examined and compared the role of TLR-4-MyD88-NF-κB pathway potentially involved in the NZB/W MCs that produced significantly more MCP-1 and OPN than the DBA/W MC controls under LPS treatment. As expected, NZB/W MCs showed a significantly enhanced activation of the TLR-4-MyD88-NF-kB pathway, although the lack of protein data for TLR-4 and MyD88 impaired interpretation of the results. In some cell types [36] and tissues [37], the effects of bacterial LPS on target cells were mediated through the TLR-4-MyD88-dependent LPS signaling pathway. Several proteins other than TLR-4 are also involved in LPS signaling. CD14 and MD-2 are helper molecules for TLR4 and are required for LPS recognition [20,23]. MyD88, an adapter molecule, is an essential component in the downstream signaling of Toll-like receptors [20,38]. We observed that NZB/W MCs showed much greater LPS-induced increases in TLR-4 mRNA and in MyD88 mRNA than the DBA/W MC controls. Activation of the TLR-4-MyD88-dependent pathway by LPS leads to the activation of NF-κB [23], an inducible transcription factor involved in cytokine-mediated inflammation [39] and in LN [40]. We detected a rapid and much greater increase in NF-κB p65 activation in NZB/W MCs after LPS stimulation compared with that in the DBA/W MC controls (Figure 4). The NF-κB inhibitors (*n*-tosyl-1-phenylalanine chloromethyl ketone and dexamethasone) markedly reduced LPS-induced MCP-1 and OPN production (Figures 5), suggesting a major role for NF-κB in NZB/W MCs that are more responsive to LPS. On the other hand, MCs also express Toll-like receptor-2 [41] and Toll-like receptor-3 [21] that caused secretion of proinflammatory cytokines. C5a receptor activation in MCs was capable of inducing proliferation, a selective production of cytokines and growth factors [42]. In the present study, we could not exclude the involvement of other signaling pathways in the activation of MCs.

The genetic basis of NZB/W mice is a complex one. In this regard, NZB/NZW mice have been extensively analyzed with respect to the genomic locations of susceptibility loci for autoantibodies, glomerulonephritis, and other component lupus phenotypes [43]. Importantly, Kikuchi and colleagues [44] reported that the NZB autoimmunity 2 (*Nba2*) locus from NZB mice is associated with autoantibody production and the subsequent development of LN. Collectively, our data support that the difference observed could be mainly encoded by the NZB genome.

Based on our data, we believe that the observed high responsiveness towards production of chemokines, such as MCP-1 and OPN, by MCs from the lupus-prone NZB/W mice (NZB/W MCs), both in the baseline (normal culture) condition and upon LPS stimulation (mimicking the LPS-induced accelerated LN model), could promote the development of the LPS-induced accelerated LN model.

## Conclusion

Our data might have clinical and pathological implications helpful in the understanding of the potential mechanism for transformation of renal lesion types from low grade to high grade in some LN patients, and helpful in the medical approach to systemic lupus erythematosus patients, associated with an incidental infection.

## Abbreviations

DBA/W = DBA-2 × NZW; ELISA = enzyme-linked immunosorbent assay; FBS = fetal bovine serum; IL = interleukin; LN = lupus nephritis; LPS = lipopolysaccharide; MC = mesangial cell; MCP-1 = monocyte chemoattractant protein-1; MHC = major histocompatibility complex; MyD88 = myeloid differentiation factor 88; NF = nuclear factor; NZB/W = NZB × NZW; OPN = osteopontin; PCR = polymerase chain reaction; TLR-4 = Toll-like receptor 4; TNF = tumor necrosis factor.

## Competing interests

The authors declare that they have no competing interests.

## Authors' contributions

S-MK performed most of the experiments and prepared the manuscript. C-WC participated in designing the primer and in statistical analysis. W-MW performed the primary MC culture. H-AS participated in the immunohistochemistry. D-MC worked on the clinical data presentation and the signaling pathway. Y-CL participated in the immunofluorescence analysis. AC was responsible for the main experimental design, data interpretation, and for finalizing the manuscript. All authors read and approved the final manuscript.
